# Failure of Neuroprotection Despite Microglial Suppression by Delayed-Start Myeloperoxidase Inhibition in a Model of Advanced Multiple System Atrophy: Clinical Implications

**DOI:** 10.1007/s12640-015-9547-7

**Published:** 2015-07-21

**Authors:** Christine Kaindlstorfer, Patrick Sommer, Biljana Georgievska, Robert J. Mather, Alan R. Kugler, Werner Poewe, Gregor K. Wenning, Nadia Stefanova

**Affiliations:** Department of Neurology, Innsbruck Medical University, Anichstraße 35, 6020 Innsbruck, Austria; Neuroscience CNSP iMed iScience, AstraZeneca R&D, Södertälje, Sweden; Neuroscience iMed, AstraZeneca R&D, Cambridge, MA USA

**Keywords:** Multiple system atrophy, Neuroinflammation, Microglial activation, Myeloperoxidase inhibition, Neurodegeneration, Synuclein

## Abstract

Multiple system atrophy (MSA) is a rapidly progressive neurodegenerative disease. Post-mortem hallmarks of MSA neuropathology include oligodendroglial α-synuclein (αSYN) inclusions, striatonigral degeneration, olivopontocerebellar atrophy, and increased microglial activation that accompanies the wide spread neurodegeneration. Recently, we demonstrated upregulation of myeloperoxidase (MPO) in activated microglia and provided evidence for the role of microglial MPO in the mediation of MSA-like neurodegeneration (Stefanova et al. Neurotox Res 21:393–404, 2015). The aim of the current study was to assess the therapeutic potency of MPO inhibition (MPOi) in a model of advanced MSA. We replicated the advanced pathology of MSA by intoxicating transgenic PLP-α-synuclein transgenic mice with 3-nitropropionic acid (3NP). After onset of the full-blown pathology, MSA mice received either MPOi or vehicle over 3 weeks. Motor phenotype and neuropathology were analyzed to assess the therapeutic efficacy of MPOi compared to vehicle treatment in MSA mice. MPOi therapy initiated after the onset of severe MSA-like neuropathology in mice failed to attenuate motor impairments and neuronal loss within the striatum, substantia nigra pars compacta, inferior olives, pontine nuclei, and cerebellar cortex. However, we observed a significant reduction of microglial activation in degenerating brain areas. Further, nitrated αSYN accumulation was reduced in the striatonigral region. In summary, delayed-start MPOi treatment reduced microglial activation and levels of nitrated αSYN in a mouse model of advanced MSA. These effects failed to impact on motor impairments and neuronal loss in contrast to previously reported disease modifying efficacy of early-start therapy with MPOi in MSA.

## Introduction

Multiple system atrophy (MSA) is a neurodegenerative disease characterized by progressive autonomic failure, cerebellar ataxia, and parkinsonism due to neuronal loss in respective brain areas (Stefanova et al. 2009; Fanciulli and Wenning 2015). Compared to idiopathic Parkinson’s disease (PD), it progresses more rapidly due to failure of levodopa benefit (Krismer et al. 2014). Mean survival is 8–10 years after symptom onset, and sudden nocturnal death is among the most frequent causes of death (Schrag et al. 2008; Shimohata et al. 2008). Currently, therapeutic interventions for MSA are limited (Kuzdas-Wood et al. 2014; Fanciulli and Wenning 2015). The lack of treatment options aiming to modify the underlying pathophysiology and the rapid deterioration of disease determine a major need for developing disease modification strategies.

To date, the underlying pathomechanisms of MSA are poorly understood, but the core feature of MSA pathology, i.e., abundant aggregates of α-synuclein (αSYN) within oligodendrocytes—also referred to as glial cytoplasmic inclusions (GCIs)—led to the idea that MSA is a primary oligodendrogliopathy linked to ectopic αSYN accumulation (Wenning et al. 2008; Song et al. 2007). The origin of αSYN in GCIs is not completely understood. Current data suggest oligodendroglial expression of the protein (Asi et al. 2014), but propagation of neuronally derived αSYN (Watts et al. 2013) may also play a role in the disease cascade of MSA. Posttranslational modifications of αSYN like hyperphosphorylation and nitration seem to associate with the abnormal inclusion formation in MSA (Duda et al. 2000; Giasson et al. 2000; Kahle et al. 2002; Fujiwara et al. 2002). GCI pathology has been associated with neurodegeneration in the nigrostriatal and olivopontocerebellar systems as well as linked to prominent microglial activation in MSA brains (Ozawa et al. 2004; Ishizawa et al. 2004).

Microgliosis and neuroinflammatory responses seem to be an important contributor to the progression of neurodegeneration in MSA; however, their roles in early stages of the disease are difficult to assess in patients. In vivo imaging demonstrates subcortical and brainstem microglial activation in the brains of MSA patients (Gerhard et al. 2003). Post-mortem analyses show constituent presence of activated microglia in MSA (Ishizawa et al. 2004, 2008; Salvesen et al. 2015). Furthermore, several signaling molecules associated with activated microglia including toll-like receptor 4 (TLR4) upregulation, NFκB nuclear translocation (Stefanova et al. 2007), and myeloperoxidase (MPO) upregulation (Stefanova et al. 2012) have been suggested to contribute to the pathogenesis of MSA and have been further addressed in experimental models. Therefore, preclinical models are instrumental to address downstream pathogenic mechanisms linked to oligodendroglial αSYN accumulation.

To model MSA, transgenic mice were generated by overexpression of human αSYN under the control of specific oligodendroglial promoters, e.g., the myelin basic protein promoter (Shults et al. 2005) or the proteolipid protein (PLP) promoter (Kahle et al. 2002) resulting in the formation of GCIs composed of insoluble, serine-129 hyperphosphorylated αSYN, resembling the hallmark pathology of the human disease. The PLP-αSYN mouse showed progressive but mild neuronal loss in substantia nigra pars compacta (SNc) and specific brainstem nuclei accompanied by microglial activation that might at least partly mediate the neuronal loss (Stefanova et al. 2005, 2007; Stemberger et al. 2010; Boudes et al. 2013; Kuzdas et al. 2013). Furthermore, PLP-αSYN mice showed increased vulnerability to oxidative stress triggered by 3-nitropropionic acid (3NP) (Stefanova et al. 2005). While both GCI-like pathology and oxidative stress alone may trigger striatonigral degeneration (SND) and both factors together result in an aggravated SND phenotype, olivopontocerebellar atrophy (OPCA) can only be initiated when both GCI-like pathology and mitochondrial dysfunction triggered by 3NP are present in the mouse brain (Stefanova et al. 2005, 2014). This feature of the combined PLP-αSYN + 3NP mouse model (further designated as MSA mouse model) makes it unique as a preclinical testbed of advanced MSA and provides the opportunity to test therapeutic interventions in a setting of widespread selective neurodegeneration representing both SND and OPCA, accompanied by GCIs and microglial activation.

Recently, we identified that MPO inhibition (MPOi) in the PLP-α-SYN mouse can suppress microglial activation and further mitigate in a dose-dependent manner the neuronal loss induced by mitochondrial dysfunction through 3NP (Stefanova et al. 2012). Our primary conclusion was that MPOi is an efficient neuroprotective strategy when applied in the early non-symptomatic stages of MSA where mild SND linked to GCI pathology precedes the aggravation of SND and the onset of OPCA triggered by 3NP. The aim of the current study was to define whether the approach of MPOi in MSA might hold promise as a neuroprotective strategy even after the onset of SND and OPCA, i.e., in a more advanced stage of the disease. We hypothesized that MPOi might reduce the level of microglial activation and post-translationally modified αSYN; however, behavioral impairments and neuronal loss may be more resistant to a delayed-start delivery in advanced MSA. For this purpose, MPO inhibition with AZD3241 or vehicle treatment was started after the induction of mitochondrial dysfunction by 3NP intoxication in PLP-αSYN mice.

## Materials and Methods

### Animals and Treatment

Homozygous transgenic PLP-αSYN mice overexpressing human αSYN under the PLP-promotor as previously characterized by Kahle and colleagues were used in this study (Kahle et al. 2002). Animals were aged 8–9 months at the beginning of the study. They were bred and housed in a temperature-controlled room under a 12/12 h (h) dark/light cycle with free access to food and water in the animal facility of the Medical University of Innsbruck under special pathogen-free conditions. During the study, observation of animal health was performed on a daily basis and all efforts were made to minimize the number of animals used and their suffering. All experiments were performed in accordance with the Austrian law and after permission for animal experiments by the Federal Ministry for Science and Research of Austria.

Transgenic male PLP-αSYN mice received 3NP intoxication according to a previously established dose escalation scheme as follows: 4 × 10, 4 × 20, 4 × 40, 4 × 50 mg/kg of 3NP, each dose applied four times intraperitoneally in a 12 h rhythm. Treatment with either AZD3241 (180 µmol/kg, *n* = 15) or vehicle (0.1 M meglumine with 20 % w/v HPbCD, *n* = 15) was commenced 1 day after the last 3NP administration and was delivered by oral gavage twice daily for 20 consecutive days in a volume of 10 ml/kg body weight. AZD3241 doses and target concentrations were chosen based on previous evidence (Stefanova et al. 2012; Tiden et al. 2011).

### Behavioral Analysis

The standardized clinical motor scale (CMS) was applied daily during the total in vivo experiment to control the efficacy of the 3NP intoxication as previously described (Fernagut et al. 2002). Evaluation was performed every morning prior to treatment throughout the study. The daily scores ranged from 0 for a healthy animal to 10 for a severely impaired mouse. For the group comparison, a mean daily score per group over the experimental time was calculated.

In addition, motor behavior was assessed at the end of the treatment period by stride length test and open field activity test as previously described (Stefanova et al. 2012) with the investigator blinded to the treatment status of the animals. For the stride length measurement, the hindlimbs of mice were wetted with nontoxic food color. The mice were let to run on a strip of paper (length: 42 cm, width: 4.5 cm) down a bright corridor to a dark enclosure. The runs for each mouse were repeated four times. Finally, the distance between consecutive steps of the left and right hindlimb was measured and a mean stride length for each mouse was determined. The initial (acceleration) and final (deceleration) 7 cm of the runs were excluded from the analysis.

The spontaneous cage activity, including the horizontal and vertical (rearing) movements, was recorded for a period of 15 min applying the FlexField activity system (San Diego Instruments, CA). The test sessions were performed in the evening in a dark noise-isolated room.

### Tissue Processing and Histological Analyses

Twenty-nine days after the start of the study, animals were sacrificed via intraperitoneal application of thiopental overdose (120 mg/kg b.w.) followed by transcardial perfusion using 4 % paraformaldehyde (PFA; pH 7.4). Subsequently, brains were quickly removed and post-fixed overnight in 4 % PFA at 4 °C. Cryoprotection was performed in a solution of 20 % sucrose in 0.01 M PBS pH 7.4 for at least 24 h. The brains were slowly frozen in 2-methylbutane at −40 °C and stored at −80 °C until further processing. Serial sections of 40 µm were cut on a cryostat (Leica, Nussloch, Germany). One full series was stained with cresyl violet (CV), according to a standard protocol. Monoclonal mouse anti-dopamine- and cAMP-regulated neuronal phosphoprotein (anti-DARPP32, BD Transduction Laboratories) was used to visualize and quantify medium-sized spiny neurons of the striatum. Monoclonal anti-tyrosine hydroxylase antibody (anti-TH, Sigma, St. Louis, Missouri) was applied to visualize and quantify nigral dopaminergic neurons. Monoclonal rat anti-mouse CD11b was used to visualize activated microglia cells (anti-CD11b, Serotec, Oxford, UK). Nitrated αSYN antibody (Invitrogen, Camarillo, CA) was used to visualize GCI-like inclusions. Secondary antibodies were biotinylated anti-mouse, or anti-rat IgG (Vector, Burlingame, CA) as appropriate. Visualization of the immunoproduct was carried out with the ABC-kit (Vectastain ABC-kit, Vector Laboratories, CA) and 3,3′-diaminobenzidine (DAB).

### Image Analysis

An observer blinded to the identity of the animals performed all stereological investigations. The optical fractionator was applied for cell number estimations in the striatum, SNc, pontine nuclei, and inferior olives using a computer-assisted image analysis system (Nikon E-800 microscope, Nikon digital camera DXM 1200; Stereo Investigator Software, MicroBrightField Europe e.K., Magdeburg, Germany). Purkinje cells were counted in a region outlined to include only the Purkinje cell layer (Stefanova et al. 2012). Microglial activation was measured by determining mean gray values of CD11b immunoreactivity in a region of interest (ROI). Background (BG) gray values were measured in the same optical field out of the section. The relative optical density (ROD) was obtained after a transformation of mean gray values by using the formula ROD = −log (mean gray ROI/mean gray BG). The density of nitrated αSYN inclusions was measured in selected brain regions by delineating the region and defining the area and the number of inclusions within. Finally, GCI density was expressed as number per mm^2^.

### Statistics

Statistical comparisons were performed with GraphPad Prism Version 5.03 using *t* test analysis to compare vehicle- and MPOi-treated groups. Repeated measures ANOVA was used to compare the progression of CMS in the vehicle- and MPOi-treated group over the period of 4 weeks. Correlations between functional measures and neuropathological readouts were done by linear regression analysis. Data in graphs are presented as mean ± standard error of the mean (SEM). *p* < 0.05 was used to determine statistical significance.

## Results

### Behavioral Analysis

**Fig. 1:**
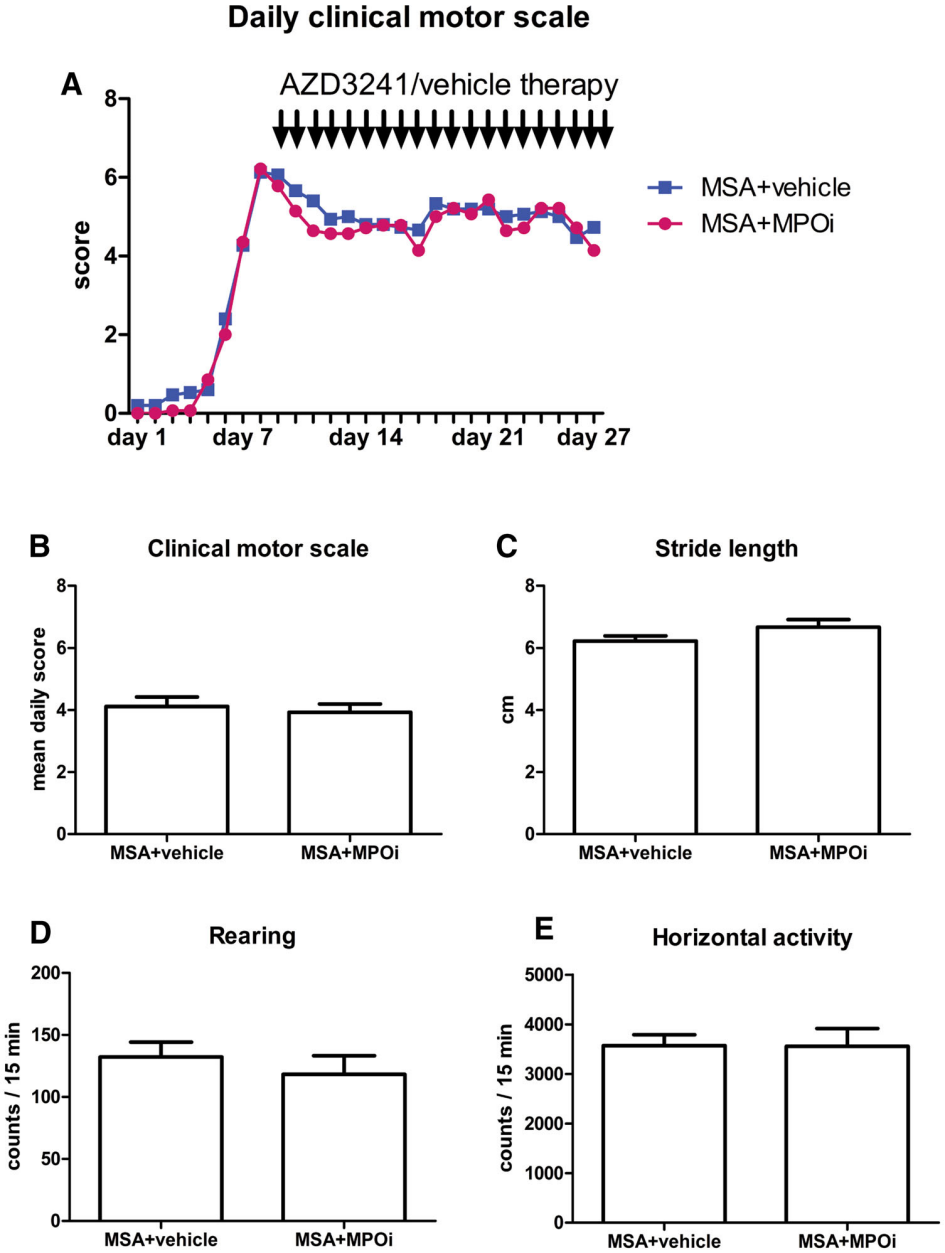
**a** The daily clinical motor score served to evaluate the time course of the motor impairment induced by 3NP treatment (day 1–day 8) and its course over the treatment period with AZD3241 or vehicle (day 9–day 30). **b** Mean clinical motor score per group over the total experimental time indicated lack of effect of AZD3241 treatment (MPOi) on the general motor disability in MSA mice. **c** Stride length was not changed under MPOi treatment of MSA mice compared to vehicle-treated ones. **d**, **e** Rearing and horizontal open field activities were not affected by the MPOi treatment of MSA mice compared to vehicle-treated MSA mice. Data are presented as mean ± SEM. MSA + vehicle group, *n* = 15, MSA + MPOi group, *n* = 14

Daily evaluation of CMS following 3NP intoxication showed progressive impairment in all animals within the first 8 days of the experiment followed by a period of disability over the next 3 weeks (effect of time: F_(3,1)_ = 143; *p* < 0.001). After day 9, when the drug treatment was initiated, the disability showed similar severity and temporal evolution in both MPOi and vehicle-treated MSA mice (effect of treatment: F_(1,3)_ = 2.05; *p* > 0.05). After 20 consecutive days of treatment with MPOi, no significant treatment effect associated with MPOi therapy could be detected (Fig. 1a, b). A similar lack of effect of MPOi on motor performance as determined by stride length (Fig. 1c) and open field activity (Fig. 1d, e) was evident at the end of the treatment period.

### Neuropathology

**Fig. 2:**
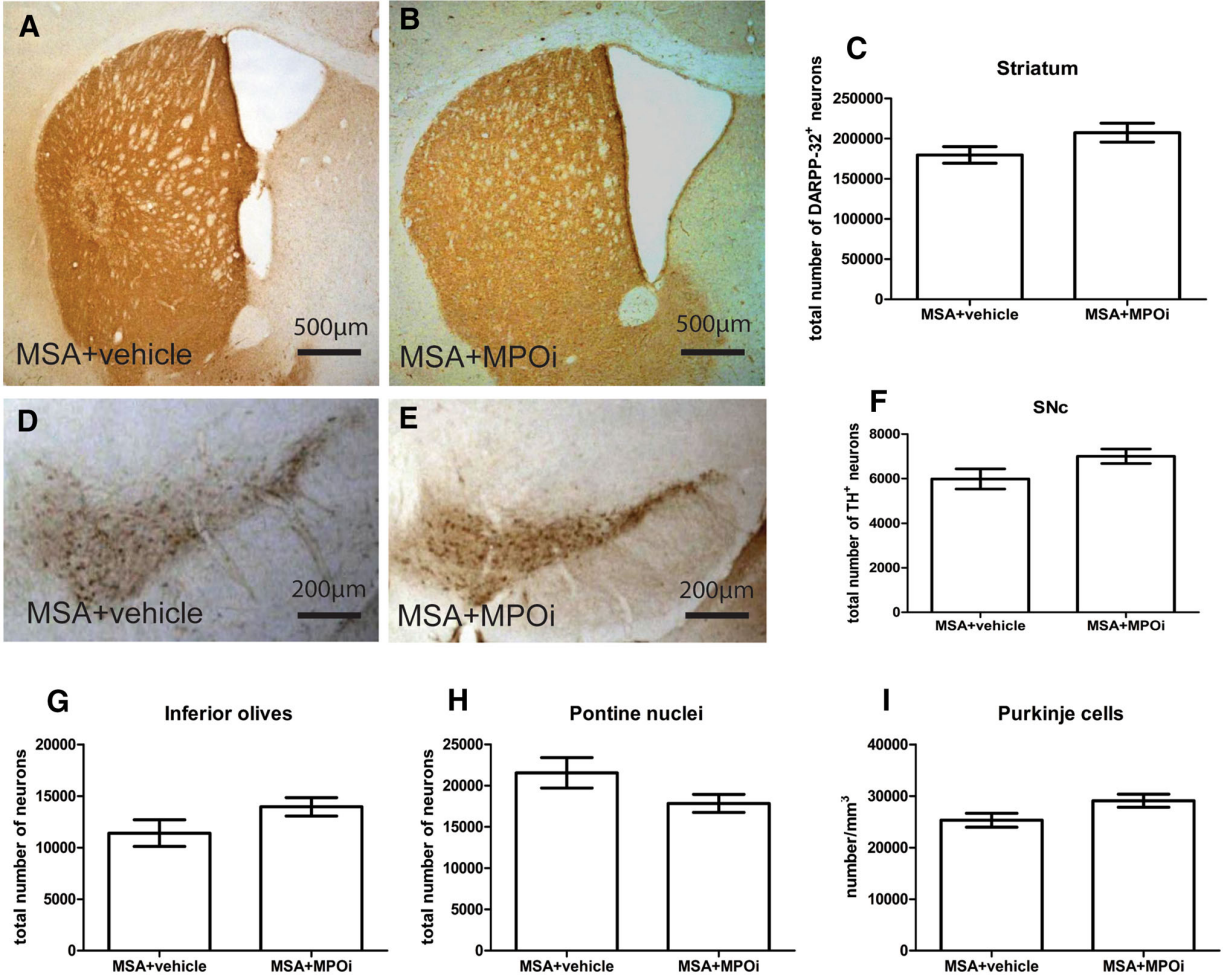
DARPP32-positive medium spiny neurons of the striatum of MSA + vehicle (*n* = 9) (**a**) and MSA + MPOi group (*n* = 7) (**b**). There was no significant effect of AZD3241 treatment on the number of striatal DARPP32 positive neurons in MSA mice (**c**). TH-positive dopaminergic neurons in SNc of MSA + vehicle (*n* = 14) (**d**) and MSA + MPOi group (*n* = 13) (**e**). MPOi treatment showed no significant neuroprotective effect on nigral TH neurons in MSA mice (**f**). Further, no neuroprotective efficacy of MPOi could be registered in the inferior olives (*n*_vehicle_ = 6, *n*_MPOi_ = 6) (**g**), the pontine nuclei (*n*_vehicle_ = 5, *n*_MPOi_ = 7) (**h**), and the Purkinje cells in the cerebellar cortex (*n*_vehicle_ = 6, *n*_MPOi_ = 8) (**i**). Data are presented as mean ± SEM

**Fig. 3:**
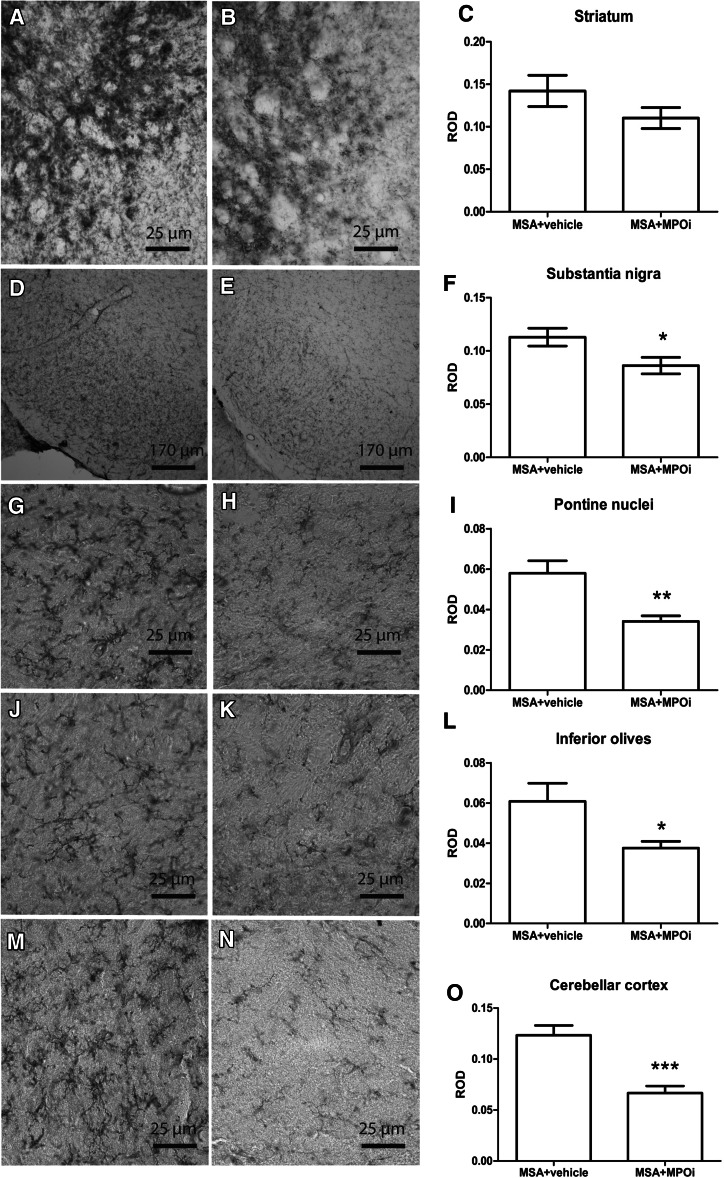
CD11b immunohistochemistry in striatum of **a** MSA + vehicle and **b** MSA + MPOi (AZD3241) group. **c** Statistical analysis indicated a tendency to reduction of the microglial activation in the striatum without reaching significance (*n*_vehicle_ = 14, *n*_MPOi_ = 14). CD11b immunohistochemistry in substantia nigra (SN) of **d** MSA + vehicle and **e** MSA + MPOi group. **f** MPOi treatment of MSA mice resulted in significant reduction of CD11b ROD in substantia nigra (*p < 0.05 compared to MSA + vehicle; *n*_vehicle_ = 14, *n*_MPOi_ = 14). CD11b immunohistochemistry in the pontine nuclei of **g** MSA + vehicle and **h** MSA + MPOi group. **i** MPOi treatment of MSA mice resulted in significant reduction of CD11b ROD in the pontine nuclei of MSA mice receiving AZD3241 compared to vehicle-treated animals (**p < 0.01; *n*_vehicle_ = 12, *n*_MPOi_ = 12). CD11b immunohistochemistry in the inferior olives of **j** MSA + vehicle and **k** MSA + MPOi group. **l** MPOi treatment of MSA mice resulted in significant reduction of CD11b ROD in the inferior olives of MSA mice receiving AZD3241 compared to vehicle-treated animals (*p < 0.05; *n*_vehicle_ = 13, *n*_MPOi_ = 14). CD11b immunohistochemistry in the cerebellar cortex of **m** MSA + vehicle and **n** MSA + MPOi group. **o** MPOi treatment of MSA mice resulted in significant reduction of CD11b ROD in the pontine nuclei of MSA mice receiving AZD3241 compared to vehicle-treated animals (***p < 0.001; *n*_vehicle_ = 11, *n*_MPOi_ = 11). Data are presented as mean ± SEM

**Fig. 4:**
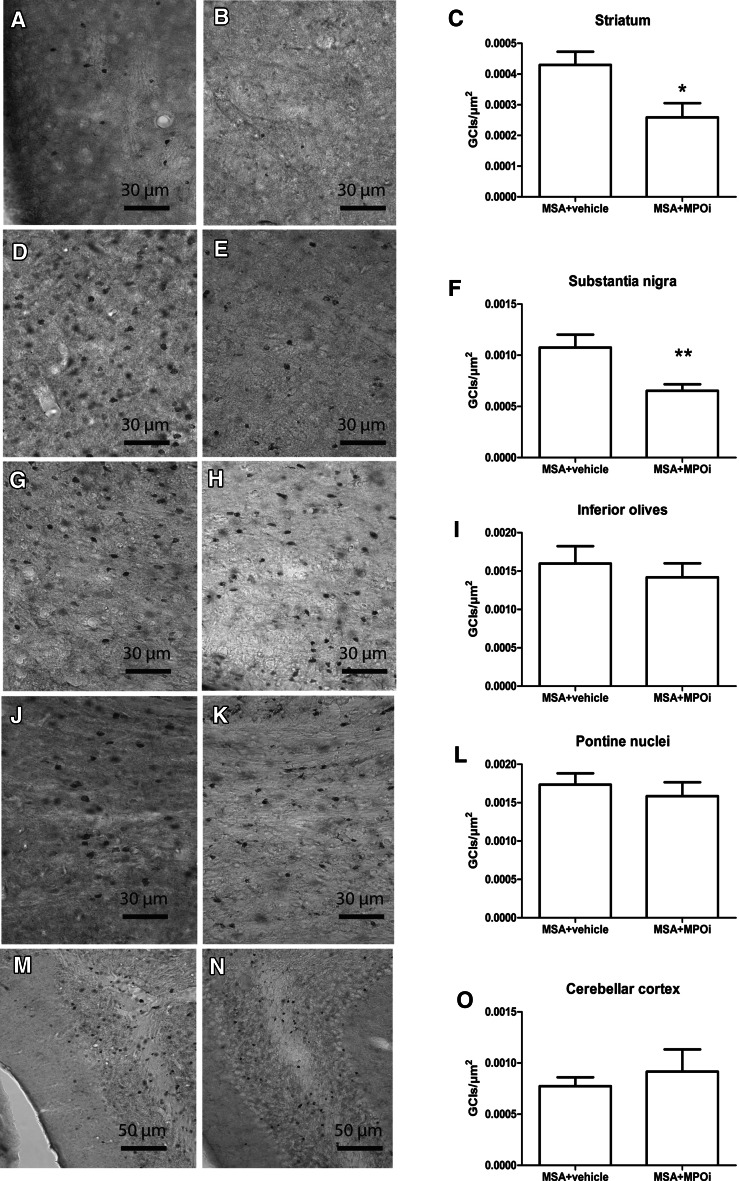
Nitrated α-synuclein immunohistochemistry in striatum of **a**, MSA + vehicle and **b** MSA + MPOi (AZD3241) group. **c** Statistical analysis indicated significant reduction of the density of GCIs positive for nitrated α-synuclein in the striatum of MSA mice receiving AZD3241 (*p < 0.05; *n*_vehicle_ = 10, *n*_MPOi_ = 13). Nitrated α-synuclein immunohistochemistry in substantia nigra of **d** MSA + vehicle and **e** MSA + MPOi group. **f** MPOi treatment of MSA mice resulted in significant reduction of GCIs density in substantia nigra (**p < 0.01 compared to MSA + vehicle; *n*_vehicle_ = 10, *n*_MPOi_ = 14). Nitrated α-synuclein immunohistochemistry in the pontine nuclei of **g** MSA + vehicle and **h** MSA + MPOi group. **i** MPOi treatment of MSA mice resulted in no significant reduction of GCIs density in the pontine nuclei of MSA mice receiving AZD3241 compared to vehicle-treated animals (*n*_vehicl*e*_ = 12, *n*_MPOi_ = 12). Nitrated α-synuclein immunohistochemistry in the inferior olives of **j** MSA + vehicle and **k** MSA + MPOi group. **l** MPOi treatment of MSA mice resulted in no change in the GCIs density in the inferior olives of MSA mice receiving AZD3241 compared to vehicle-treated animals (*n*_vehicle_ = 13, *n*_MPOi_ = 12). Nitrated α-synuclein immunohistochemistry in the cerebellar cortex of **m** MSA + vehicle and **n** MSA + MPOi group. **o** MPOi treatment of MSA mice resulted in no change in the GCIs density in the cerebellar cortex of MSA mice receiving AZD3241 compared to vehicle-treated animals (*n*_vehicle_ = 11, *n*_MPOi_ = 11). Data are presented as mean ± SEM

To assess the efficacy of MPOi treatment in a model of advanced MSA, we measured neuronal numbers in SNc, striatum, pontine nuclei, inferior olives, and cerebellar cortex (Purkinje cells). Neuronal numbers remained unaffected by the MPOi treatment compared to vehicle in all studied regions (Fig. 2). However, a strong biological effect of the MPOI treatment was detected on microglial activation being significantly reduced in SNc (*p* = 0.027), pontine nuclei (*p* = 0.0018), inferior olives (*p* = 0.02), and corpus callosum (*p* = 0.0056). There was significant correlation between the levels of microglial activation and the number of nigral neurons (*R*^2^ = 0.1686, *p* = 0.0334). Although there was a numerical decrease in the ROD of microglial activation in the striatum after MPOi treatment (MSA + vehicle, 0.14 ± 0.018 vs. MSA + MPOi, 0.11 ± 0.012), the difference to vehicle-treated mice did not reach statistical significance (*p* = 0.1632) (Fig. 3). Furthermore, the treatment with MPOi resulted in significantly reduced density of nitrated αSYN inclusions compared to vehicle-treated MSA mice in SNc (*p* = 0.0022) and striatum (*p* = 0.016) but not in the inferior olives (*p* = 0.47), pontine nuclei (*p* = 0.53), or the cerebellar cortex (*p* = 0.55) (Fig. 4).

## Discussion

We provide preclinical evidence that MPO inhibition in a model of advanced MSA suppresses microglial activation without significant preservation of neurons in the striatonigral or the olivopontocerebellar regions of the brain and no significant behavioral changes compared to vehicle-treated MSA mice. Furthermore, we report significant reduction of nitrated αSYN-positive inclusions in SNc and striatum but not in the olivopontocerebellar pathway, suggesting that nitrated αSYN accumulation in the MSA mouse brain is not exclusively determined by levels of microglial activation. Finally, our findings suggest that the reduction of microglial activation induced by MPOi (as confirmed previously both preclinically (Stefanova et al. 2012) and in a clinical trial (Jucaite et al. 2014)) may interfere with the degree of neuronal loss, and the degree of GCI density and motor improvement; however, these effects are significantly detectable only by early-start or “preventive” delivery of MPOi in a model of “early” MSA (Stefanova et al. 2012) but masked in a delayed-start paradigm applied to a model of “advanced” MSA.

Microglial activation is a prominent and consistent feature of human MSA pathology (Ishizawa et al. 2004; Gerhard et al. 2003; Salvesen et al. 2015) and its contribution to the disease mechanisms is broadly discussed (Fellner et al. 2011; Halliday and Stevens 2011; Sanchez-Guajardo et al. 2015). At post-mortem examination, microglial burden in the degenerating gray matter does not correlate with the remaining neuronal counts (Ishizawa et al. 2008); however, it is still unclear what the role of microglial activation in earlier stages of the disease may be and to what extent it contributes to the neurodegenerative process. Experimental evidence suggests that microglial activation in MSA models may have detrimental role through the upregulation of iNOS and increased release of nitric oxide in SNc in early stages of the disorder (Stefanova et al. 2007). Alternatively subpopulation of activated microglial cells may be responsible for the clearance of deleterious αSYN species through TLR4, thus having a rescue effect for nigral neurons (Stefanova et al. 2011). The current study together with our previous observations on the role of MPOi in early MSA (Stefanova et al. 2012) suggests that microglial activation can have different degree of effect on neuronal loss in MSA, dependent on the disease stage. When microglial activation is suppressed in early stages of the disease through MPOi, significant neuroprotection can be observed (Stefanova et al. 2012). Microglial suppression through the same dose and mode of application of MPOi in an advanced stage of neuronal loss in MSA mice is ineffective to protect the degenerating neurons in the affected areas. The current observations point toward the paramount importance of identifying an optimal treatment window in relation to the time course of neurodegeneration, which may explain the lack of efficacy of the delayed-start MPOi treatment on neuronal loss and motor function. However, we identified mild but significant correlation between levels of microglial activation and number of nigral neurons, supporting previously reported neuroprotection linked to reduced microglial activation.

Furthermore, nitric oxide and superoxide released by activated microglia have been previously proposed as mediators that link neuroinflammation and abnormally nitrated αSYN accumulation which may finally lead to nigral neuronal loss (Gao et al. 2008). Our results here support this conclusion indicating that suppression of microglial activation by MPOi treatment indeed leads to significant reduction of nitrated αSYN accumulation in SNc and striatum as we showed also in a previous experiment (Stefanova et al. 2012). However, no other of the analyzed regions—inferior olives, pontine nuclei, and cerebellar cortex—showed significant interrelations between the level of microglial activation and the density of nitrated αSYN inclusions suggesting region-specific interactions between oxidative stress, microglial activation, and αSYN pathology in a model of advanced MSA. Region-specific vulnerability has been previously described in PLP-αSYN mice. In this transgenic model, SNc and striatum undergo progressive neurodegeneration induced by GCI-like pathology (Stefanova et al. 2014) and this pathology can be further aggravated by mitochondrial dysfunction (3NP). On the contrary, the olivopontocerebellar region can be affected by neurodegeneration only in the presence of both GCIs and mitochondrial dysfunction (3NP) (Stefanova et al. 2005). This region-specific vulnerability is now further complemented by the observation that the interaction between mitochondrial dysfunction and GCIs can be modulated by microglial activation in the striatonigral but not in the olivopontocerebellar region.

As mitochondrial dysfunction is a valid candidate pathogenic mechanism in MSA as suggested by the finding of Coq2 mutations in MSA families (The Multiple-System Atrophy Research Collaboration 2013) and the central role of αSYN in the pathogenesis of MSA is unequivocal, it seems that the combined PLP-αSYN + 3NP model provides a relevant tool to address these two factors, their interplay and interaction with neuroinflammatory responses. It is likely that the subacute injury with 3NP in the PLP-αSYN mouse differs from the slowly progressive human MSA pathology which may relate to a better efficacy of MPOi therapy in human MSA. Despite the limitations of the mechanistic modeling approach that cannot 100 % reflect the dynamics of the human disease, we believe that it provides important insights into the possible scenarios of interaction between GCI pathology, mitochondrial dysfunction, and neuroinflammation and can give relevant answers to their role in development of therapeutic strategies for MSA.

### Clinical Relevance

The current dataset provides an important follow-up on the preclinical screening of MPOi and its therapeutic efficacy dependent on the stage of treatment initiation in a model of MSA. We reported previously that a preventive therapeutic regimen of MPOi in MSA mice replicating human GCI-like pathology prevented motor deterioration and conferred neuroprotection in SNc, striatum, cerebellar cortex, pontine nuclei, and inferior olives (Stefanova et al. 2012). The neuroprotective effects of MPOi were associated with reduction of microglial activation and diminished density of GCIs in the affected brain areas. Despite the profound beneficial behavioral and neuroprotective effects of MPOi therapy when initiated in a presymptomatic phase of MSA, our current results show that the effects of the therapy may be limited when drug application is started in an advanced symptomatic stage of the disease. Although we observed reduced microglial activation correlating with mild nigral neuronal preservation, this was not associated with significant motor improvement or reversal of SND and OPCA pathology in a double-hit model of advanced MSA.

In line with our results and their clinical relevance, a recent report on AZD3241 treatment in PD patients showed reduced levels of [^11^C]PBR28 binding to dopamine transporter and translocator protein in PET imaging indicating suppressed microglia activity (Jucaite et al., 2014). Taken together, the findings on MPOi therapy in the MSA mouse model and the randomized controlled trial in PD patients unequivocally point toward a strong biological effect of MPOi in the CNS and indicate that early timing of delivery is paramount in future MSA trials.
